# Diabetes, Endothelial Dysfunction, and Vascular Repair: What Should a Diabetologist Keep His Eye on?

**DOI:** 10.1155/2015/848272

**Published:** 2015-05-18

**Authors:** V. Altabas

**Affiliations:** Department for Endocrinology, Diabetes and Metabolic Diseases “Mladen Sekso”, Clinic for Internal Medicine, University Hospital Center “Sestre Milosrdnice”, 10000 Zagreb, Croatia

## Abstract

Cardiovascular complications are the most common complications of diabetes mellitus. A prominent attribute of diabetic cardiovascular complications is accelerated atherosclerosis, considered as a still incurable disease, at least at more advanced stages. The discovery of endothelial progenitor cells (EPCs), able to replace old and injured mature endothelial cells and capable of differentiating into healthy and functional endothelial cells, has offered the prospect of merging the traditional theories on the pathogenesis of atherosclerosis with evolving concepts of vascular biology. The literature supports the notion that EPC alterations are involved in the pathogenesis of vascular diseases in diabetics, but at present many questions remain unanswered. In this review the aspects linking endothelial progenitor cells to the altered vascular biology in diabetes mellitus are discussed.

## 1. Introduction

Cardiovascular complications are the most common and devastating complications of diabetes mellitus; they are a major cause of hospital admissions and leading cause of death among diabetic patients [1, 2]. A prominent attribute of diabetic cardiovascular complications is accelerated atherosclerosis, which is associated with oxidative stress, insulin resistance, and the metabolic syndrome [3, 4]. Current knowledge suggests that endothelial injury and dysfunction occur as the initial event in the pathogenesis of atherosclerosis, followed by platelet adhesion and aggregation [5]. Overproduction of cytokines and other inflammation mediators stimulates migration and proliferation of smooth muscle cells in the vascular intima and deposition of extracellular matrix molecules like collagen and elastin, leading to plaque expansion and fibrous cap formation [6, 7]. Fibrous caps may weaken and rupture eventually, exposing the underlying extremely thrombogenic tissues. Plaque rupture induces further thrombus formation and release of more inflammatory mediators, causing continued progression of the atherosclerotic plaque, finally resulting in luminal narrowing and/or occlusion. Dramatic events like myocardial infarction, ischemic stroke, or critical ischemia of peripheral tissues may appear, depending of the anatomic site of the injured vessel [8, 9]. With increasing knowledge about the pathogenesis of atherosclerosis, hope that human atheromata can regress has evolved, but over time this idea met considerable skepticism. Resistance to concepts of lesion regression was enhanced by the fact that advanced atheromata in humans and in animals contain necrosis, calcification, and fibrosis, giving an impression of still irreparable events [10–13].

The discovery of a cell subgroup of myeloid origin, able to replace old and injured mature endothelial cells and capable of differentiating into healthy and functional endothelial cells, challenged skeptics. Those cells, named endothelial progenitor cells (EPC), have offered the prospect of merging the traditional theories on the pathogenesis of atherosclerosis with evolving concepts of atherosclerosis regression. Indeed, it seems that these progenitor cells are able to repair the injured vessel wall and to enhance neovascularization of ischemic tissues [14–16].

On the contrary, reduced EPC levels are associated with more serious endothelial dysfunction and elevated risk of adverse cardiovascular events, compatible with the concept that impaired EPC-mediated vascular repair allows further progression of vascular disease [17].

This applies in particular to endangered patients with metabolic alterations such as compensatory hyperinsulinemia, impaired fasting glucose, impaired glucose tolerance, and diabetes, who have an impaired EPC number and function, and this could be a further challenge to future investigations [14, 18].

However, available data suggest that metabolic interventions by either lifestyle change, better glucose and lipid control, or certain other agents are able to improve EPC number and function [17].

This review will focus on the role of EPC in vascular repair and available therapeutic options in diabetic patients.

## 2. Endothelium Biology

Despite being originally considered to be just a simple mechanic barrier between the blood and vascular wall, the endothelium is now recognized as the most important component of healthy vascular function. It maintains the anticoagulant, antiplatelet, and fibrinolytic properties of vascular cells. The healthy endothelium in response to physical and chemical signals produces a wide range of factors that regulate vascular tone, cellular adhesion, thromboresistance, smooth muscle cell proliferation, and vessel wall inflammation. In few words, the endothelium is regarded as a very complex endocrine and paracrine organ [19, 20].

Effects on the vascular tone were the first discovery unveiling the importance of the endothelium. The endothelium produces several vasoactive molecules that relax or constrict the vessels, interplaying with circulating vasoactive mediators like bradykinin or thrombin. This vasomotion is of crucial importance for tissue oxygen supply and metabolic demand and is also involved in remodeling of vascular structures and regulating long-term organ perfusion. Maintaining the functional integrity of the endothelium, therefore, is critical for the preservation of blood flow and the prevention of thrombosis [21, 22].

Nitric oxide (NO) is the most important mediator released by endothelial cells, historically named and originally identified as endothelium-derived relaxing factor. NO is produced from L-arginine by the action of endothelial NO synthase (eNOS) in the presence of several cofactors. This gas activates guanylate cyclase in vascular smooth muscle cells, leading to cGMP-mediated vasodilatation. In addition, this enzyme may be activated under certain circumstances by other signaling molecules like bradykinin, adenosine, vascular endothelial growth factor (during hypoxia), and serotonin (during platelet aggregation) [23, 24].

The endothelium also mediates vasodilatation through other mechanisms, like the endothelium-derived hyperpolarizing factor via accumulation of potassium ions in the intercellular space and/or due to tissue electrical conductivity, allowing propagation of electrical signals along the axis of blood vessels by means of homocellular gap junctions and throughout the vascular wall itself by means of myoendothelial gap junctions [25, 26].

In normal vascular physiology, NO plays a key role in preserving the vessel wall in a quiescent state by inhibition of inflammation, thrombosis, and cellular proliferation through limiting oxidative phosphorylation in mitochondria and s-nitrosylation of cysteine residues in a wide range of proteins, including transcription factors like NF*κ*B, cell cycle-controlling proteins, and proteins involved in generation of tissue factors [22, 27].

Another endothelium-derived vasodilator that acts independently of NO is prostacyclin, derived by the action of the cyclooxygenase system. Prostacyclin is an eicosanoid which chiefly prevents formation of the platelet plug involved in primary hemostasis. In humans, it appears to have a more limited role in the maintenance of vasodilator tone, although it may contribute to some of the other regulatory roles of the endothelium [22, 26].

Other substances important for vasomotion are constrictors like endothelin and vasoconstrictor prostanoids generated in the endothelium, as well as angiotensin I converted to angiotensin II at the endothelial surface. These vasoconstrictor agents predominantly act locally but may also exert some systemic effects and have a role in the regulation of arterial structure and remodeling. Because of these properties, vasoconstrictor substances are believed to be involved in the pathogenesis of vascular diseases of several organ systems, including the heart, general circulation, and brain [22, 28, 29].

## 3. Diabetes, Endothelial Injury, and Dysfunction

Chronic hyperglycemia leads to vascular disease, and multiple studies in patients and animal models and in vitro have revealed that hyperglycemia alters endothelial metabolism and function, causing vascular injury. Vascular injury contributes to all diabetic complications, whether micro- or macrovascular, in all forms of diabetes mellitus. Prolonged and/or repeated exposure to other cardiovascular risk factors can additionally seriously damage the endogenous protective mechanisms within endothelial cells. As a consequence, the endothelium may become dysfunctional, and lose is vasomotor properties [30, 31].

An important biochemical abnormality accompanying diabetes mellitus and also important for vascular injury is the formation of advanced glycation end products (AGEs). Driven by hyperglycemia and oxidant stress, the effects of AGEs on vessel wall homeostasis may account for the rapidly progressive atherosclerosis associated with diabetes [32, 33].

Although the mechanisms underlying this phenomenon are probably multifactorial, the role of the diacylglycerol-protein kinase C (PKC) pathway has recentyl been recognized as very important in in-vivo and in-vitro studies. PKC may have several adverse effects on endothelial function, such as activation of superoxide-producing enzymes like the nicotinamide adenine dinucleotide phosphate (NADPH) oxidase, and increased expression of a dysfunctional, superoxide-producing, uncoupled endothelial nitric oxide synthase (NOS III). PKC-mediated superoxide production may inactivate NO derived from endothelial NOS III and may inhibit the activity and/or expression of the NO downstream target, the soluble guanylyl cyclase, resulting in impaired vasomotion properties of the endothelium. Furthermore, within the vessel wall, collagen-linked AGEs may “trap” plasma proteins, interact with specific receptors, and modulate a large number of cellular properties. On plasma low density lipoproteins (LDL), AGEs initiate oxidative reactions that promote the formation of oxidized LDL [32–35].

However, in patients with diabetes, endothelial dysfunction is a coherent finding. There is a general consensus that hyperglycemia and diabetes lead to impaired NO production and damaged vasodilatatory activity [33, 35].

Even more importantly, endothelial cells exposed to chronic hyperglycemia can also lose integrity, progress to senescence, and undergo apoptosis [36, 37].

The outcome of this detrimental process is detachment of endothelial cells, which are released into the circulation. In the bloodstream they can be detected as circulating mature endothelial cells or as their apoptotic microparticles, if endothelial cells did not detach as entire cells [38].

McClung et al. have shown that circulating mature endothelial cell levels are higher in type 2 diabetic patients, irrespective of glucose control represented by HbA_1c_ levels. Elevated endothelial cell-derived endothelial microparticle levels are predictive of the presence of coronary heart disease, and it is an even more significant independent risk factor than presence of diabetes, lipid levels, or hypertension [38–40].

The result of this apoptotic process is arterial denudation, which triggers a cascade of proatherosclerotic processes like platelet adhesion and aggregation, inflammation, smooth muscle cell proliferation, migration, and matrix secretion [38, 41].

## 4. Endothelial Repair

As already mentioned, structural or functional damage of the endothelium is a result of cumulative exposure to cardiovascular risk factors. These factors have the ability to induce biochemical cellular toxicity and/or promote endothelial cell loss by apoptosis.

The resulting extent of endothelial dysfunction depends not only on the extent of injury, but also on the biologic capacity for repair. For this reason, endothelial reparatory mechanisms are crucial in reestablishing vessel integrity.

Two mechanisms involved in this process have been recently identified.

The endothelium itself has a relatively weak capacity for self-repair, because it is built from mostly terminally differentiated cells with low proliferative capacity. However, mature endothelial cells surrounding the injured locus in the endothelium can replicate in situ and replace lost and damaged cells [38, 42].

Recently, it has become evident that some forms of cells, recruited from the bone marrow and from some other tissues, circulate in the peripheral blood and have the ability to be embedded in the injured endothelium and differentiate into mature cells with endothelial characteristics. These cells were called EPCs, and they seemed to be an important mechanism for maintenance and repair of the endothelium.

Several studies have shown that EPCs may be derived from different sources such as the bone marrow and non-bone marrow organs like the spleen. EPCs are a heterogeneous group of cells presenting in different stages of differentiation in the blood stream. There are at least two different known subtypes of EPCs: the early and late EPCs. Early EPCs occur as colony forming units (CFU) and have some endothelial characteristics, like harboring markers of CD31, TIE2, and VEGFR2. Late EPCs, or outgrowth EPCs, have different growth patterns. Outgrowth EPCs express additional endothelial characteristics, such as VE-cadherin and von Willebrand factor, in addition to CD31, CD133, CD34, and VEGFR2 [4]. These outgrowth EPCs will further differentiate into mature endothelial cells for angiogenesis and vasculogenesis [14, 38, 43]. The ability of EPCs for functional vascular repair varies with the maturation state of the cell [14, 38].

There are some difficulties in precise characterization of EPCs, because some of these cell surface markers are shared with other cell types like hematopoietic stem cells and mature endothelial cells. Currently, EPCs are defined as cells positive for both a hematopoietic stem cell marker such as CD34 and an endothelial marker protein such as VEGFR2 [14, 38, 44].

Mobilization of these cells into the circulation is in a certain magnitude NO-dependent and may be impaired in patients with present cardiovascular risk factors, like diabetes and smoking [45]. There are also several factors responsible for mobilizing EPCs from the bone marrow or other organs into the blood stream, like growth factors and cytokines, including VEGF (vascular endothelial growth factor), SDF-1*α* (stromal-cell-derived factor-1*α*), erythropoietin, and estrogens. Interestingly, the number of EPCs seems to be inversely correlated with the severity and degree of atherosclerosis [46, 47].

EPCs migrate toward injured endothelial regions, after they have been mobilized into the circulation. At these places they home or adhere and start to proliferate beginning vascular repair. An important factor in directing circulating progenitor cells to sites of vascular injury is chemokine signaling, like tissue hypoxia induced upregulation of SDF-1*α* in ischemic tissues. Homing to the injured sites is facilitated through interactions between SDF-1*α* and CXCR4 (CXC chemokine receptor 4) [48–50].

Once embedded in the injured site, EPCs are involved in endothelial repair either by proliferation and forming new endothelial cells or by releasing provasculogenic cytokines and growth factors important for the proliferation of local mature endothelial cells or other EPCs [48].

Recent evidence has suggested that cardiovascular risk factors interfere not only with the differentiation and function of endothelial progenitor cells, but also with the recruitment of these cells [14, 38, 39, 44, 45].

However, blood flow in ischemic tissues could be enhanced by the increase in the number of endothelial circulating progenitor cells, augmented whether by cell transfusion or induced mobilization, involving mechanisms like enhanced restoration and integrity of the endothelial lining, and neointimal formation [46, 51, 52].

## 5. Endothelial Repair in Diabetic Patients

Diabetes, hypercholesterolemia, hypertension, and smoking, leading to atherosclerosis, and several other forms of vascular disease are associated with a reduced number and impaired functional activity of circulating EPCs [14, 18, 31, 32, 38].

Apparently, there is a glucometabolic continuum in EPC biology. In particular, all forms of glucose disorders are associated with abnormalities in EPC biology, including impaired fasting glucose and impaired glucose tolerance [47].

Individuals with disorders of glucose metabolism have reduced levels of circulating EPCs. Damaged mobilization, decreased proliferation, and shortened survival in the circulation may contribute to a reduced number of circulating EPCs in diabetic patients. Several mechanisms could be responsible for defects in EPC mobilization, migration and homing of EPC in diabetic patients, including decreased NO bioavailability, defects in intracellular signaling, inflammation and adipokines, reactive oxygen species, and direct effects of insulin and IGF-1 [38, 53, 54].

NO bioavailability and PI3K/Akt signaling are crucial in EPC mobilization from bone marrow. In insulin resistant states both mechanism are damaged [53, 55].

A consistent finding in insulin resistant humans and animals with impaired EPC mobilization and function in experimental models is decreased NO bioavailability. NO is important for the normal function of EPCs after mobilization. A substantial requirement for NO in migration, homing, and neovascularization was shown in in vitro and in vivo studies [53, 55, 56].

Another important mechanism damaged in insulin resistant humans and animals is the PI3K/Akt pathway. This pathway mediates metabolic effects like insulin-stimulated glucose uptake in metabolically active tissues and insulin- or shear-stress induced NO production in endothelial cells. In EPCs, inhibition of this pathway abolishes their mobilization in response to several stimuli [47].

After mobilization, there are more issues limiting the regenerative ability of EPCs in diabetes.

The capacity for effective homing to the injured blood vessel, adhesion and integration into the endothelium, proliferation, and differentiation is crucial for EPCs to promote vascular repair. EPCs from diabetic animals and humans show impaired response to chemotactic stimuli, reduced proliferative potential, and diminished ability to form vascular-like structures in vitro. Hyperglycemia is associated with reduced expression of SDF-1*α*, a chemokine occurring in injured tissues, and decreased expression of CXCR4 in peripheral mononuclear cells and EPCs. Both may inhibit homing of EPCs from the circulation to the injured endothelium, since adhesion of EPCs to sites of vascular injury is dependent on an interaction between locally produced chemokines and the CXCR4 receptor [48, 49].

Damaged PI3K/Akt signalling has been implicated to impair EPC differentiation and inhibition of EPC apoptosis, like in inflammatory states [57]. Systemic inflammation is known to contribute to atherosclerotic vascular disease by stimulation of proatherogenic adhesion molecules in mature endothelial cells, but it affects also EPC-mediated vascular regeneration. Inflammatory factors impair EPC survival, differentiation, and function. On the contrary, inflammatory mediators released from the injured endothelium stimulate the production of growth factors and cytokines necessary to facilitate EPC release and homing. It is likely that persistent inflammation (even a low level inflammation) has harmful effects, but transient inflammatory response following endothelial injury is associated with EPC mobilization and may be beneficial [47, 58].

Adipocytes are another factor involved in damaged vascular repair in diabetic patients. These metabolic active cells produce several cytokines and contribute to a chronic inflammatory state by secreting the proinflammatory cytokine TNF-*α* which reduces the proliferation of EPCs [59].

Leptin, another adipokine, increased in vitro tubule formation in EPC cultures, but at higher concentration EPC migration was inhibited. Leptin was also shown to be able to enhance the capacity of EPC to adhere to matured endothelial cells or the extracellular matrix by increasing the expression of specific integrins. These effects led to enhanced reendothelialization when leptin-stimulated human EPCs were given to mice. However, obese insulin resistant humans have increased circulating leptin concentrations and impaired leptin effects and have been assumed to suffer from “leptin resistance” [60, 61].

Reactive oxygen species (ROS) produced in diabetic patients also contribute to endothelial injury and impair endothelial repair. ROS are directly cytotoxic for endothelial cells, react with NO, decrease NO bioavailability, and form peroxynitrite anions which act as powerful oxidants. Although it seems that EPCs from healthy individuals are relatively resistant to oxidant stress, in diabetic patients ROS may lead to EPC impairment. Mechanisms may include increased ROS production and impaired endogenous antioxidant defence [62, 63].

Among other substances, insulin and IGF-1 influence the mobilization and differentiation of EPCs. In a small study of patients with poorly controlled type 2 diabetes, insulin therapy led to an increase in circulating EPCs. Insulin-mediated EPC mobilization was significantly dependent on SDF-1 polymorphism; mobilization was significantly enhanced in subjects with the SDF-1 3′-A/G allele. Insulin stimulates the clonogenic and angiogenic potential of EPCs; the effect is more likely mediated through the IGF-1 receptor than the insulin receptor itself. Humpert and coworkers have demonstrated that insulin stimulates the outgrowth in vitro of EPCs from patients with type 2 diabetes. This effect was completely abrogated by IGF-1 receptor blockade but unaltered after blocking the insulin receptor itself. The IGF-1 receptor-dependent effect of insulin on EPC growth was largely mediated by the MAPKs ERK1/2 (extracellular-signal-regulated kinase) and p38 [64, 65].

## 6. Current Available Therapeutic Options

New concepts of endothelial injury and repair have offered exciting perspectives for new researches in preventing and/or treating cardiovascular disease. Despite the fact that EPCs are recently discovered, lifestyle interventions and several drugs successfully used for treatment of diabetes and/or cardiovascular diseases have proven beneficial effects to EPC biology.

Many of those lifestyle and pharmacological interventions already have established favourably cardiovascular benefits [14, 17].

Exercise as a lifestyle modification has a potential to increase the number of endothelial progenitor cells and improve their migratory capacity, helping to repair the damaged endothelium [66–68]. In healthy volunteers, exercise increased the number of EPCs in the circulation [69]. In patients with stable coronary artery disease, an increased number of circulating EPCs and reduced EPC apoptosis were found after 28 days of moderate exercise training [70].

Vascular repair could be enhanced also with nutrition. There is some evidence for favourable effects of polyunsaturated fatty acids from different sources on vascular biology [71–73]. A hypocaloric Mediterranean diet has also been proved as enhancing endothelial repair [74, 75].

Considering current available medical treatment for diabetes and its comorbidities, there are several established classes of drugs with beneficial effects on endothelial repair.

Among these drugs are statins, angiotensin-converting enzyme inhibitors, angiotensin II type 1 receptor blockers, some sulphonylureas like gliclazide, metformin, PPAR-gamma agonists, GLP-1, DPP-4 inhibitors, and insulin, which are able to increase the number and/or functional activity of EPCs, affecting different mechanisms, as some experimental and clinical studies have suggested.

Statins are widely used among diabetic and nondiabetic patients as antilipemic drugs, with a profound effect on the incidence of cardiovascular events and mortality. They may also modulate vascular repair through increasing EPC numbers and inducing EPC differentiation by activation of the PI3K pathway and stimulation of NO production, in addition to their primary effects on lipoprotein metabolism [57, 76].

In general, the effect of statins on EPC count was a significant increase occurring shortly after treatment initiation. It seems that it could be a class effect of all statins, since there are many of them studied with similar results, including simvastatin, atorvastatin, pravastatin, and rosuvastatin. Some secondary outcomes like left ventricular (LV) volume, ejection fraction (EF), and flow mediated dilation (FMD) could also be improved in some studies. Furthermore, even herbal products containing lovastatin have the capacity to improve EPC counts. Only one study showed a negative correlation between statin therapy and EPC count, but this study was cross-sectional, on a group of patients receiving various statins. It is still unclear whether the effect of statins is dose dependent, and larger randomized trials are necessary for firmer proof.

An overview of clinical data involving statins treatment and their effects on EPCs is shown in Table 1.

Similarly, beneficial outcomes on EPC biology were found for some antihypertensive drugs with favourable metabolic effects, like ACEIs (angiotensin-converting enzyme inhibitors) and ARBs (angiotensin II type 1 receptor blockers). For both classes of drugs cardiovascular benefits are well established, beyond their antihypertensive effect [94]. It was shown that ACEi and ARB treatment increased numbers and function of EPCs in patients with a variety of cardiovascular diseases, including arterial hypertension, stable coronary artery disease, and acute coronary syndromes. Treatment with these classes of antihypertensive drugs could contribute to their cardioprotective effect in type 2 diabetes, even independent of their action on blood pressure [95] like in a study where both olmesartan and irbesartan increased the numbers of EPCs in a group of patients with type 2 diabetes.

Calcium channel blockers (CCBs) decrease blood pressure by inhibiting L-type voltage-gated calcium channels, leading to a decreased level of intracellular calcium. They act on vascular smooth muscle, inducing vasodilation followed by decreased blood pressure. Preliminary results from two studies reported affirmative outcomes on EPC numbers and function with CCBs in patients with essential hypertension [96, 97].

In addition to data on EPC, in some trials there are also data about improved flow mediated vessel dilatation, markers of inflammation, and in one trial there are also favorable mortality data. Most trials presented affirmative results in regard to EPCs count and/or function, with few exceptions. However, larger trials are needed to get more reliable data.

A summary of currently available clinical data on antihypertensives and their effect on EPC biology is shown in Table 2.

Several classes of agents used for treatment of diabetes are also shown to enhance EPC biology. Among them is metformin, commonly used as first-line treatment in patients with type 2 diabetes and as supplementary treatment in patients with type 1 diabetes. It has also established vasculoprotective effects. These effects could be at least in part explained by increased circulating EPCs after initiating treatment with metformin [107, 108]. The effect was even augmented with addition of gliclazide [108]. Otherwise, positive effects of gliclazide as monotherapy could be also proven; it improved also flow mediated vessel dilatation and some markers of oxidative stress [109].

A further class of antidiabetic medications found to have beneficial effects on vascular repair are thiazolidinediones. Thiazolidinediones are peroxisome proliferator-activated receptors (PPAR) *γ* agonists and are in clinical use for glucoregulation, but these insulin-sensitizing drugs could also improve some other cardiovascular risk factors. At the time, pioglitazone and rosiglitazone are approved for use by the FDA. Considering EPC biology, rosiglitazone was able to normalize impaired EPC migratory activity and to increase EPC numbers in culture [117]. In patients with type 2 diabetes rosiglitazone was effective in reducing NADPH oxidase activity and thus improving the reendothelialization by EPCs [63]. Pioglitazone was also shown to increase the number and function of EPCs and to decrease EPC apoptosis in animal models [118]. Human studies with pioglitazone were also mainly affirmative [110–113].

Other mechanisms in glucose control affect glucagon like peptide 1 (GLP-1) and its analogues and inhibitors of degradation.

GLP-1 is a hormone, released from enteroendocrine cells in the intestine, and has been shown to exert cardiovascular protective effects. Results indicated that GLP-1 improves VEGF generation, which contributed to improvement of EPCs biological function. VEGF is a necessary mediator of the effects of GLP-1 on EPCs [119]. Interestingly, there are no data about GLP injectables and EPCs number and function in diabetic patients.

Dipeptidyl peptidase 4 (DPP 4) inhibitors are a recently introduced class of oral hypoglycemic agents. There is one study that showed an increase in EPC number in patients treated with sitagliptin after 4 weeks of treatment in comparison to metformin. In addition, plasma stromal-derived factor-1*α* (SDF-1*α*) levels also increased in sitagliptin treated patients, leading to enhanced EPC release from bone marrow [114]. In another study sitagliptin improved both SDF-1*α* levels and flow mediated dilatation. In this study voglibose was used as active comparator but with no effect on EPC biology despite its positive effect on blood vessels dilation [115].

In subjects with type 1 diabetes and in patients with type 2 diabetes who fail to respond adequately to oral therapies, insulin is used to achieve glycaemic control. Although there is some evidence that short-term insulin treatment and tight blood glucose control decrease adverse cardiovascular events after myocardial infarction, studies have failed to show superiority of insulin in comparison to other drugs used for glucose control on the long term [120]. However, as discussed previously, insulin and certain insulin analogues have been shown to mobilize EPCs and improve EPC parameters in vitro [47]. In patients with type 2 diabetes mellitus, long acting insulin analogues glargine and detemir were able to raise the EPC count, with no significant difference between both drugs. Differences were noticed in the number of hypoglycemic events and weight gain, in favour of insulin detemir [116].

Studies on effects of antidiabetic treatment on EPCs are listed in Table 3.

Some other hormones occasionally used in the treatment of specific subgroups of diabetic patient may also improve vascular repair, like estrogens and erythropoietin.

Estrogens are shown to be effective in mobilizing EPCs and reducing neointima formation after arterial injury in animals [121]. These effects are NOS-mediated and depend on FGF-2 (fibroblast growth factor-2) activity. Furthermore, in healthy fertile women, EPCs are mobilized cyclically in response to raising estrogens during the menstrual cycle, providing an interesting explanation for gender differences in cardiovascular risk [47, 122].

Erythropoietin, a kidney hormone that controls erythropoiesis, was also expected to be beneficial in improving vascular repair in humans but without definitive proof yet. In a trial on patients with ST-elevation acute myocardial infarction there was only a nonsignificant improvement in EPC count after a single dose of erythropoietin in the acute phase, with no impact on infarct size [123].

## 7. Current Limitations and Future Perspectives

The discovery of circulating endothelial progenitor cells has challenged current concepts about the genesis and treatment of atherosclerosis and has unveiled very wide experimental and clinical perspectives. With respect to prominent medical, social, and economic impact of diabetic cardiovascular complications, researchers and clinicians involved in diabetology should not remain indifferent to these novel findings.

Since their discovery in 1997, accumulating findings suggest that EPCs promote postnatal vasculogenesis in adults, important for vascular repair of the injured endothelium, opening the way for new therapies of cardiovascular diseases focused on EPCs.

Despite these encouraging prospects, there are still issues and limitations that need to be addressed [124].

A special challenge is that patient groups who would gain the greatest benefit from new EPC based clinical concepts, like diabetic patients, are the same in whom different problems with decreased EPC number and their dysfunction have been clearly demonstrated [124, 125]. This problem limits approaches based on EPCs isolation, cultivation, and autologous transplantation in diabetic patients. In fact, new insights on reduced EPC counts and EPC dysfunction in diabetic patients will maybe allow us to introduce diabetic osteomyelopathy as a new, but very important, diabetic chronic complication.

Therefore, it is necessary to put more efforts into understanding mechanisms of EPC dysfunction and then to design new strategies to improve EPC function ex vivo before therapeutic transplantation.

Similar problems face other groups of patients, like elderly people. EPCs derived from aged individuals also show a reduced capacity to proliferate, home into existing capillary networks, and enhance perfusion like in diabetic patients [124–126].

Another problem is how to obtain adequate numbers of those cells. Furthermore, every autologous delivery of cells inevitably involves a considerable time delay in treatment, due to the time needed for collection, identification, isolation, and then propagation of progenitors ex vivo [124].

Adverse effects of endothelial progenitor cell delivery may include microvascular embolism and unintended acceleration of pathological neovascularization in malignancies. Undirected growth and possible undesired pathological differentiation after transplantation of those cells may generate risks of late complication, like teratoma. In fact, vigorous differentiation and purification protocols are needed, and studies proving the long-term safety profile of progenitor cells are required before widespread use in humans [124].

It seems to be more likely that a greater impact on general health could have concepts focused on prophylactic measures like lifestyle changes and/or pharmacological interventions with drugs specially designed to address EPC proliferation, migration, and homing to injured vessels.

## 8. Conclusion

Studies have proven the prognostic significance of endothelial function, which is most often clinically demonstrated as the vasodilator response to various pharmacological or mechanical stimulations. Endothelial dysfunction may occur over time, progressing to atherosclerotic plaques and clinical apparent vascular disease.

Since endothelial injury and dysfunction precede clinically significant atherosclerotic vascular disease and play a role in its pathogenesis, the discovery of EPCs has provided a new concept of vascular disease as potentially preventable and curable, offering new strategies for medical intervention.

Since EPCs are of extreme importance for reendothelialization of the injured endothelium, promoting vascular repair may be an attractive therapuetic approach. Maintaining normal EPC numbers and function seems to be crucial in preventing cardiovascular diseases.

There are still unresolved questions and many challenges to face before EPC based therapies will be widely used, but even with contemporary medical interventions the number and function of EPC may be improved. This is especially important for patients with metabolic alterations such as compensatory hyperinsulinemia, impaired fasting glucose, and IGT, whose EPC number is decreased and function is impaired.

## Figures and Tables

**Table 1:** 

Reference	Study drug	Study population and design	Study duration	Findings
Vasa et al. [77]	Atorvastatin 40 mg/day	15 patients with coronary artery disease, no control group	4 weeks	1.5-fold ↑ in EPC count after 1 week 3-fold ↑ in EPC count after 4 weeks ↑ EPC functional activity

del Papa et al. [78]	Simvastatin 20 mg/day	40 patients (20 hypercholesterolemic versus 20 normocholesterolemic patients with systemic sclerosis)	4 weeks	Simvastatin ↑ EPC count in patients without systemic sclerosis In patients with systemic sclerosis there was an attenuated response, mainly in patients with late disease

Westerweel et al. [79]	Simvastatin 80 mg/day versus simvastatin 10 mg/ezetimibe 10 mg/day	20 obese patients with metabolic syndrome, randomized trial, crossover design	6 + 6 weeks	↑ EPC counts in both groups

Pesaro et al. [80]	Simvastatin 80 mg/day versus simvastatin 20 mg/ezetimibe 10 mg/day	68 patients with LDL levels >70 mg/dL pretreated with simvastatin 20 mg, randomized trial	6 weeks	No effect on EPC count in either group Similar reduction in LDL levels in both groups

Hibbert et al. [81]	Atorvastatin 80 mg/day versus no statin	20 male patients undergoing angiography for stent placement randomized to atorvastatin or no statin treatment	4 days	3.5-fold ↑ in EPC count in the statin group

Baran et al. [82]	Atorvastatin 40 mg/day versus placebo	60 patients undergoing first-time CABG, placebo controlled, randomized double-blind study	14 days	↑ EPC count in atorvastatin group ↓ incidence of postoperative atrial fibrillation in the statin group

Sobrino et al. [83]	Atorvastatin 20 mg/day	48 patients with first ever nonlacunar ischaemic Stroke 16 patients receiving atorvastatin during the first 4 days	7 days	↑ EPC count in atorvastatin group Effect probably due to NO related mechanisms

Huang et al. [84]	Atorvastatin 40 mg/day versus atorvastatin 10 mg/day	100 patients with ischemic cardiomyopathy randomized to 10 mg or 40 mg of atorvastatin Control group: 100 healthy volunteers	1 year	40 mg of atorvastatin had a more profound ↑ in EPC count Higher dose of atorvastatin was associated with a more marked ↓ in total and LDL cholesterol, hsCRP, oxLDL, and circulating endothelial microparticles

Spadaccio et al. [85]	Atorvastatin 20 mg/day versus placebo	50 patients undergoing elective coronary surgery, randomized crossover trial	3 weeks	↑ EPC count in atorvastatin group SDF-1*α*, CSF, and VEGF unaffected

Leone et al. [86]	Atorvastatin 80 mg/day immediately versus 20 mg/day atorvastatin after hospital discharge	40 patients with AMI undergoing PCI, randomized trial	4 months	Larger dose of atorvastatin related to larger ↑ EPC count LV volume, EF, and wall motion were similar in both groups after study completion

Lu et al. [87]	Pravastatin versus placebo versus Xuezhikang	88 patients with essential hypertension, randomized trial	8 weeks	↑ EPC count and proliferative ability in pravastatin and Xuezhikang (contains lovastatin) group

Paradisi et al. [88]	Pravastatin 40 mg/day	20 patients, healthy postmenopausal women, randomized, double-blind trial	8 weeks	↑ EPC colony forming units ↓ count of senescent cells

Tousoulis et al. [89]	Rosuvastatin 10 mg/day	60 patients with systolic heart failure, randomized trial	1 month	↑ EPC count improved No change in inflammatory and oxidative markers

Erbs et al. [90]	Rosuvastatin 40 mg/day versus placebo	42 patients with chronic heart failure, randomized trial	12 weeks	↑ EPC count ↑ homing of EPC ↑ FMD, ↑VEGF

Yoshida et al. [91]	Pitavastatin 2 mg/day versus placebo	30 male smokers, randomized trial	4 weeks	No effect on EPC count ↑ FMD, ↓ markers of oxidative stress in pitavastatin group

Spiel et al. [92]	Simvastatin 80 mg/day versus rosuvastatin 10 mg/day versus placebo	6 healthy volunteers, randomized, double-blind, placebo controlled, crossover study	5 days	3-fold ↑ EPC count in statin groups Class effect?

Hristov et al. [93]	Low dose of statins (10/20 mg/day) versus high dose (40 mg/day) versus untreated	209 CAD patients (without statin: 65, low dose statin: 101, and higher dose statin: 43 patients) cross-sectional study	None	40 mg/d of statin treatment has significantly ↓ EPC count Lower doses had no impact on EPC count Continuous statin therapy inversely correlated with EPC numbers

**Table 2:** 

Reference	Study drugs	Study population and design	Study duration	Findings
Cacciatore et al. [98]	Enalapril 20 mg/day versus zofenopril 30 mg/day,	36 patients with newly diagnosed mild hypertension, randomized trial	5 years	↑ EPC count No difference between groups ↓ intima media thickness

Sun et al. [99]	Perindopril 4 mg/day versus placebo	68 patients with acute myocardial infarction and T2DM	28 days after PCI	↑ EPC count ↑ VEGF ↑ SDF-1*α* ↑ LVF ↓CV mortality in the perindopril group

Min et al. [100]	Ramipril 5 mg/day	36 nondiabetic patients with acute myocardial infarction	4 weeks	↑ EPC count 1.5-fold after 1 week, 2.5-fold after 4 weeks ↑ EPC proliferation, migration, and adhesion

Cangiano et al. [101]	Perindopril 10 mg/day versus valsartan 320 m/day	Patients with acute coronary syndromes 16 receiving perindopril 17 receiving valsartan 20 healthy controls	30 days	↑ EPC mobilization, ↑ VEGF in the perindopril group No effects found for valsartan

Porto et al. [102]	Ramipril 5 mg/day versus telmisartan 80 mg/day	42 patients with acute coronary syndrome, randomized trial	20 days after PCI	↑ EPC count in both groups Telmisartan had a more profound anti-inflammatory effect

Pelliccia et al. [103]	Telmisartan 40 mg/day versus placebo	40 normotensive patients with CAD, randomized trial	4 weeks	↑ EPC count ↑ FMD

Bahlmann et al. [95]	Olmesartan 40 mg/day versus placebo, double-blind RCT Irbesartan 300 mg/day, open trial	18 patients with T2DM randomized to olmesartan or placebo 20 patients with T2DM receiving irbesartan	12 weeks	↑ EPC count with both olmesartan and irbesartan

Tan et al. [104]	Losartan 100 mg/day			↑ EPC count ↑ FMD

Suzuki et al. [105]	Losartan 50 mg/day versus trichlormethiazide 4 mg/day	36 patients with hypertension randomized to losartan or trichlormethiazide Control group: 18 normotensive patients	4 weeks	↑ EPC count with losartan Hypertensive patients had a lower EPC count in comparison to normotensive patients

Kampoli et al. [106]	Pioglitazone (15 m/day) versus perindopril (4 mg/day)	50 patients with T2DM, randomized trial	1 month	No effect on EPC

Sugiura et al. [96]	Nifedipine SR 20 mg/day versus placebo	37 hypertensive patients with stage I hypertension, randomized trial	4 weeks	↑ EPC count ↑ EPC differentiation migration, resistance to oxidative stress ↑ FMD

de Ciuceis et al. [97]	Barnidipine 20 mg/day versus hydrochlorothiazide 25 mg/day	29 hypertensive patients with mild essential hypertension, randomized trial	6 months	↑ EPC count with barnidipine No difference in RR reduction observed between drugs

**Table 3:** 

Reference	Study drug	Study population and design	Study duration	Study findings
Chen et al. [109]	Gliclazide 30–90 g/day	33 patients with newly diagnosed T2DM versus 25 nondiabetic patients in the control group	12 weeks	↑ EPC count ↑ flow mediated dilatation ↓ some markers of oxidative stress in study group

Chen et al. [108]	Gliclazide (30–60 g/day) and metformin (250–1000 mg/day) versus metformin (500–2500 mg/day)	47 patients with newly diagnosed T2DM, randomized trial	16 weeks	more profound ↑ EPC count and function with combination treatment

Liao et al. [107]	Metformin (1700–2550 mg/day)	46 patients with newly diagnosed T2DM versus 51 healthy controls	16 weeks	↑ EPC count in both groups T2DM patients had a lower EPC count throughout the study ↑ FMD changed in both groups

Werner et al. [110]	Pioglitazone 45 mg/day versus placebo	54 patients without T2DM, with stable CAD, randomized trial	30 days	↑ EPC count ↑ migratory activity of EPCs ↑ clonogenic potential of EPCs after pioglitazone treatment

Wang et al. [111]	Pioglitazone 30 mg/day	24 patients with T2DM receiving pioglitazone versus 12 patients with T2DM receiving metformin, randomized trial	8 weeks	↑ EPC count and homing and decreased ↓ EPC apoptosis, ↓ hsCRP, ↓ triglycerides, ↓ LDL, ↑ HDL cholesterol, and ↑ insulin sensitivity after pioglitazone treatment No change in FMD

Makino et al. [112]	Pioglitazone (15–30 mg/day)	34 patients with T2DM	24 weeks	↑ EPC count ↑adiponectin ↓ hsCRP

Esposito et al. [113]	Pioglitazone (15–45 mg/day) versus metformin (1000–2000 mg/day)	110 patients with newly diagnosed T2DM, randomized trial	24 weeks	More profound ↑ EPC count ↑ weight ↑ HDL ↑ adiponectin ↓ CRP ↓ triglycerides in patients receiving pioglitazone

Kampoli et al. [106]	Pioglitazone (15 m/day) versus perindopril (4 mg/day)	50 patients with T2DM, randomized trial	1 month	No effect on EPC count Improved markers of inflammation and oxidative stress

Fadini et al. [114]	Sitagliptin 100 mg/day versus no additional treatment	16 patients with T2DM receiving sitagliptin, 16 patients with T2DM with no additional treatment, controlled, nonrandomized trial	4 weeks	↑ EPC count ↑ SDF-1*α* in the study group

Nakamura et al. [115]	Sitagliptin (50 m/day) versus voglibose (0,6 mg/day)	66 patients with T2DM, 31 patients with T2DM, receiving sitagliptin, 35 patients with T2DM receiving voglibose	12 weeks	↑ EPC count with sitagliptin ↑ FMD in both groups, no difference between groups

Fadini et al. [116]	Insulin detemir versus insulin glargine	42 patients with T2DM and macroangiopathy, randomized crossover study	6 months	↑ EPC count increased between month 3 and month 6 in both groups ↑ weight gain and ↑ hypoglycemic events with glargine
